# Reverse transcriptase inhibitors prevent liver abscess formation during *Escherichia coli* bloodstream infection

**DOI:** 10.1073/pnas.2319162121

**Published:** 2024-01-16

**Authors:** Karthik Hullahalli, Katherine G. Dailey, Yuko Hasegawa, Welkin E. Johnson, Matthew K. Waldor

**Affiliations:** ^a^Department of Microbiology, Harvard Medical School, Boston, MA 02115; ^b^Division of Infectious Disease, Brigham and Women’s Hospital, Boston, MA 02115; ^c^Biology Department, Boston College, Chestnut Hill, MA 02467

**Keywords:** liver abscess, bloodstream infection, endogenous retroviruses, reverse transcriptase inhibitors

## Abstract

The presence of bacteria in the bloodstream is associated with severe clinical outcomes. In mice, intravenous inoculation of *Escherichia coli* can lead to the formation of macroscopic abscesses in the liver. Abscesses are regions of severe necrosis and consist of millions of bacteria surrounded by inflammatory immune cells. Liver abscess susceptibility varies widely across strains of mice, but the host factors governing this variation are unknown. Here, we profiled hepatic transcriptomes in mice with varying susceptibility to liver abscess formation. We found that transcripts from endogenous retroviruses (ERVs) are robustly induced in the liver by *E. coli* infection and ERV expression positively correlates with the frequency of abscess formation. Hypothesizing that ERV-encoded reverse transcriptase may generate cytoplasmic DNA and heighten inflammatory responses, we tested whether nucleoside/nucleotide reverse transcriptase inhibitors (NRTIs) influence abscess formation. Strikingly, a single NRTI dose administered immediately following *E. coli* inoculation prevented abscess formation, leading to a concomitant 100,000-fold reduction in bacterial burden. We provide evidence that NRTIs inhibit abscess formation by preventing the tissue necrosis that facilitates bacterial replication. Together, our findings suggest that endogenous reverse transcriptases drive inflammatory responses during bacterial bloodstream infection to drive abscess formation. The high efficacy of NRTIs in preventing abscess formation suggests that the consequences of reverse transcription on inflammation should be further examined, particularly in infectious diseases where inflammation drives negative clinical outcomes, such as sepsis.

Intravenous (IV) inoculation of C57BL mice with *Escherichia coli* leads to the formation of liver abscesses (1). Our observations suggest a model where inflammatory responses drive abscess formation by creating a replicative niche for *E. coli*. Following inoculation, inflammatory immune cells infiltrate the liver, leading to necrosis of liver tissue. Bacteria within the necrotic regions replicate, resulting in further immune cell recruitment and abscess formation. Within this framework, diminishing inflammatory responses is expected to reduce development of abscesses; indeed, mice lacking TLR4, the membrane-bound receptor for lipopolysaccharide, do not develop abscesses.

Other strains of mice such as BALB/cJ, 129S1/SvImJ, and CBA/J have functional TLR4 alleles but are resistant to abscess formation (1). Furthermore, within C57BL mice, we observed differences in abscess susceptibility across substrain and sex; for example, only among females, C57BL/6N (B6N) mice are more susceptible than C57BL/6J (B6J) mice. Backcross analysis revealed that multiple B6J loci control abscess susceptibility (1). These observations underscore the complex and multifaceted nature of the mechanisms that underlie abscess formation. Here, focusing solely on female mice, we set out to identify host factors that drive abscess susceptibility.

## Results and Discussion

### Expression of Endogenous Retroviruses Correlates with Abscess Susceptibility.

**Fig. 1.**
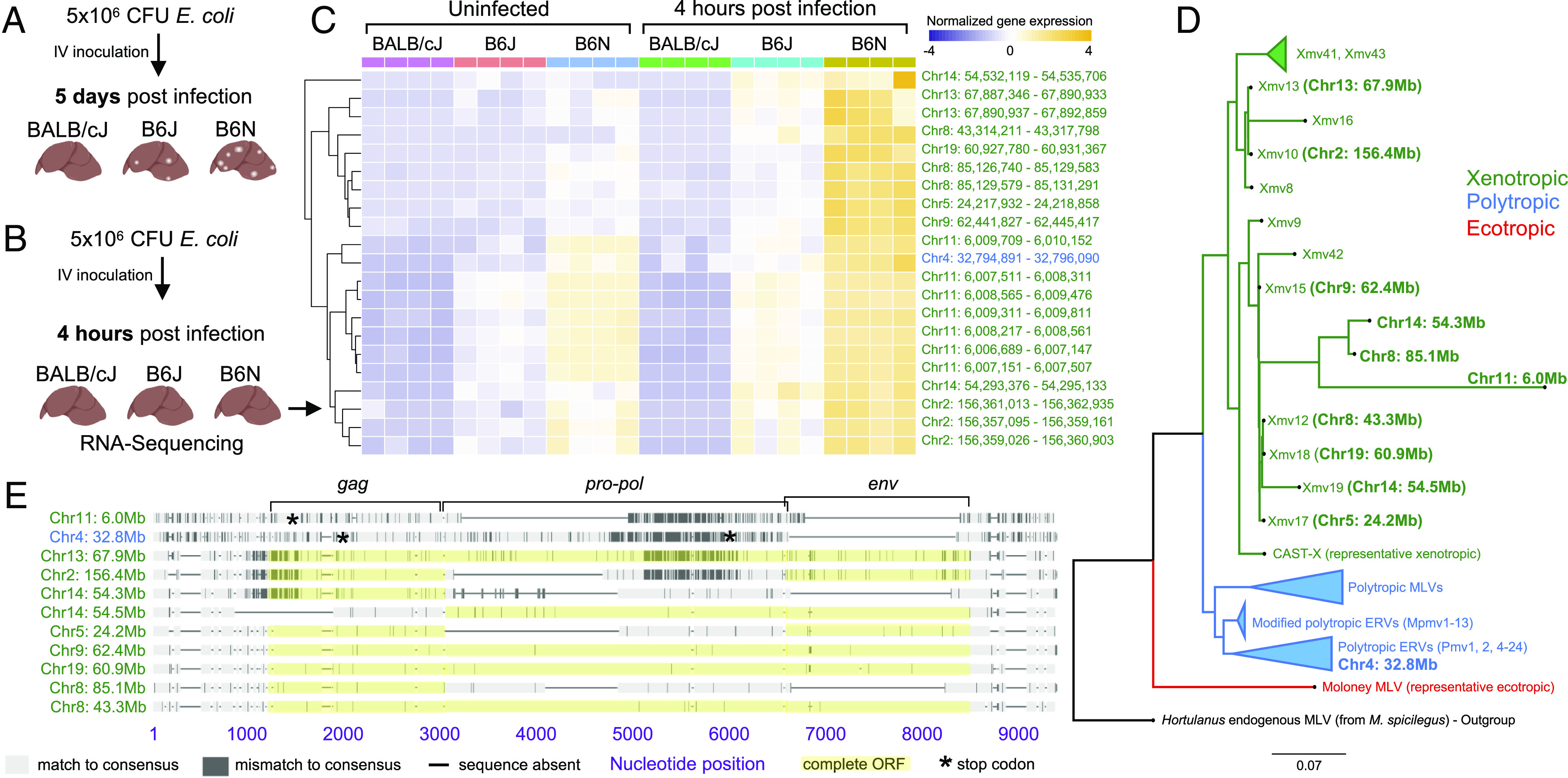
ERV expression correlates with abscess susceptibility. (*A*) Schematic of variation in abscess susceptibility in BALB/cJ, B6J, and B6N female mice. (*B*) Schematic of RNA-Sequencing experiment. (*C*) Z-score normalized expression of transcripts that are induced by infection in B6N mice and whose expression correlates with abscess susceptibility. The complete dataset, including non-ERV genes, is in Datasets S1 and S2. Coordinates are from GRCm38. (*D*) Maximum likelihood tree of abscess-associated and representative ERV loci in B6J. Loci identified in this study are in bold and those identical to previously known loci are in parentheses [e.g., Xmv13 (Chr13: 67.9 Mb)]. Four loci lack *env* and do not match previously known ERVs (3). For clarity, one xenotropic and several polytropic MLVs are represented as single, collapsed branches (triangles). (*E*) Sequence alignment of the 11 abscess-associated MLV loci compared to their consensus.

Following IV *E. coli* inoculation, ~90% of B6N female mice develop liver abscesses while BALB/cJ mice are entirely resistant. B6J females display an intermediate susceptibility, where abscesses are less numerous and less likely to develop, leading to fewer colony-forming units (CFU) in B6J relative to B6N [Fig. 1*A*, (1)]. To identify host factors that control abscess formation, we performed RNA-sequencing on livers of BALB/cJ (resistant), B6J (intermediate), and B6N (sensitive) females at 4 hours post inoculation (hpi), a time point prior to bacterial replication but when inflammatory responses are triggered (Fig. 1*B*). We reasoned that abscess-promoting genes would be up-regulated following infection, more highly expressed in B6N relative to B6J, and more highly expressed in B6J relative to BALB/cJ. These criteria (*P*-adj < 0.05, log2 FC > 1) identified 13 genes, but none appeared to function together in common pathways (Dataset S1). However, two genes (Gm42031 and Gm43305) mapped to endogenous retroviruses (ERVs). By reexamining all retroelements in the gEVE database (2), including many loci not present in the initial analysis, we identified 21 transcripts derived from 11 distinct ERVs whose expression positively correlated with abscess susceptibility (Fig. 1*C* and Dataset S2).

ERVs are integrated proviruses from past retroviral infections and have coevolved with eukaryotic genomes for millennia (4). Functional ERVs can be transcribed, packaged, and secreted as mature viral particles, as well as reverse-transcribed to DNA and reintegrated into the chromosome. Mice possess several classes of ERVs that reflect varying histories of viral infections (5). Comparative analysis of the 11 abscess-associated ERV loci against known murine ERVs revealed that 10 loci cluster with xenotropic murine leukemia viruses (MLV) and related ERV loci (*Xmvs*), while one clusters with polytropic MLVs and related ERV loci (*Pmvs*) (Fig. 1*D*). Four loci encode full-length proviruses with intact reading frames for all viral proteins (Fig. 1*E*), including the major structural polyprotein (Gag), reverse transcriptase/integrase (Pol), and envelope protein (Env). These data raise the possibility that *E. coli* bloodstream infection leads to the production of MLV particles and/or the production of cytosolic DNA.

### Nucleoside/Nucleotide Reverse Transcriptase Inhibitors Prevent Abscess Formation.

ERV reverse transcription can generate cytosolic DNA, which can induce immune signaling through cytoplasmic nucleic acid sensors (6). We hypothesized that reverse transcriptases drive abscess formation by stimulating inflammatory responses via creation of cytosolic DNA. To assess the role of reverse transcription in abscess formation, we treated B6N mice with nucleoside/nucleotide reverse transcriptase inhibitors (NRTIs), which are also used to treat and prevent HIV infection (6, 7).

**Fig. 2.**
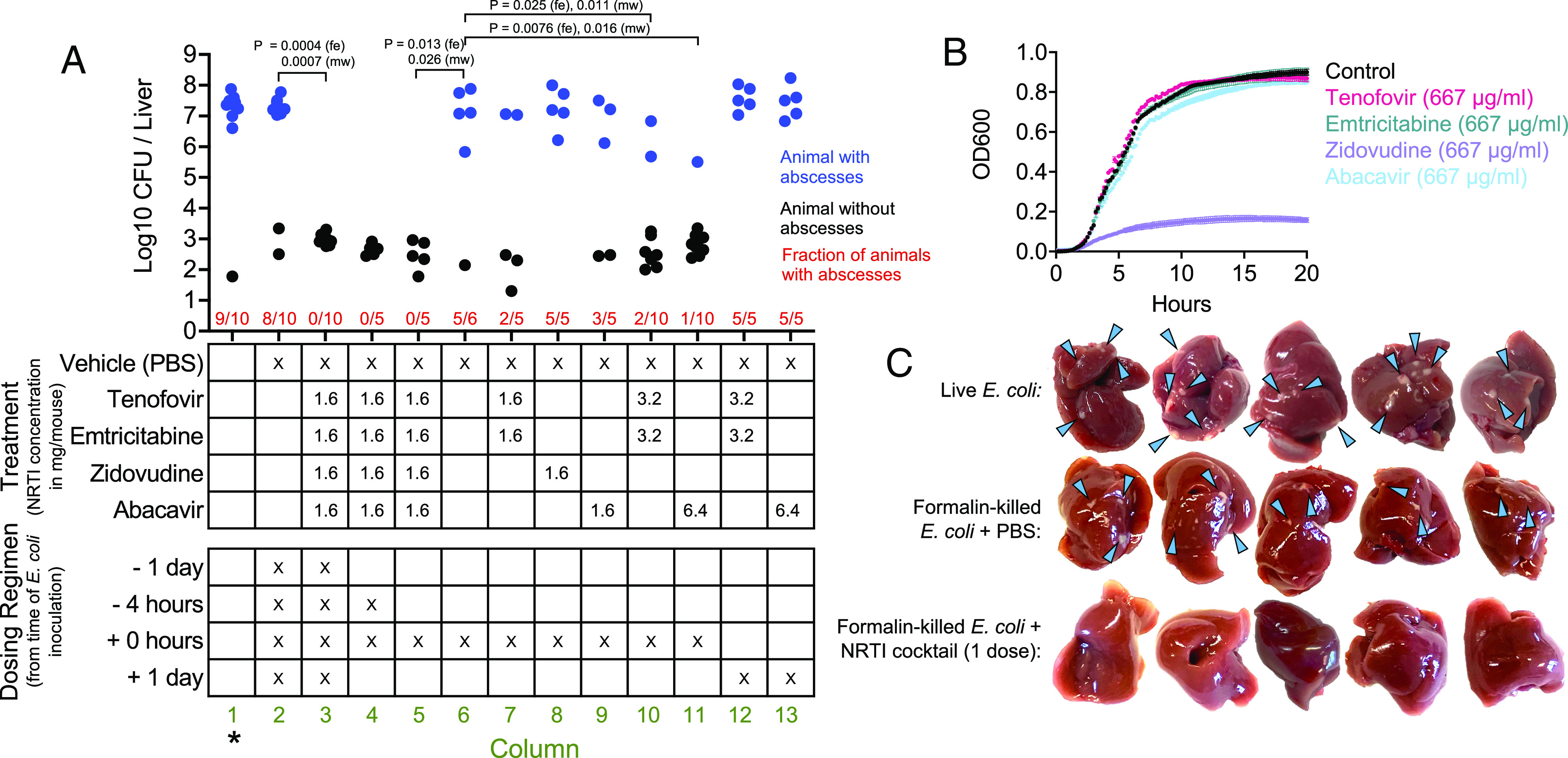
NRTI treatment prevents abscess formation. (*A*) B6N females were inoculated IV with *E. coli* and NRTIs or vehicle control was injected IP at indicated times (*Bottom* table) and doses (*Top* table). CFUs are shown per liver, along with whether the animal possessed (blue) or lacked (black) abscesses, as well as the fraction of animals with abscesses (red). *P* values are derived from one-tailed Mann–Whitney *U* tests (mw, to compare CFUs) or Fisher Exact tests (fe, to compare the fraction of animals that developed abscesses). *historical data from ref. 1 for reference. (*B*) Growth curves of *E. coli* exposed to NRTIs in LB (n = 4, mean ± SD). (*C*) Effect of NRTI treatment (all four drugs IP, one dose immediately following inoculation) on the formation of necrotic lesions caused by formalin-killed *E. coli* (*Middle* and *Bottom* rows, blue arrows). Abscesses formed from inoculation with live *E. coli* are shown for reference (*Top* row, blue arrows).

Abscess formation was entirely prevented when a cocktail consisting of 1.6 mg each of tenofovir, emtricitabine, zidovudine, and abacavir was administered intraperitoneally 1 d prior to inoculation, 4 h prior to inoculation, immediately following inoculation, and 1 d post inoculation (dpi) (Fig. 2*A*, column 3). The high efficacy of the NRTI cocktail was also observed with a single dose administered immediately following *E. coli* inoculation (Fig. 2*A*, column 5). To determine whether any individual constituent of the cocktail was responsible for preventing abscess formation, we treated mice with tenofovir/emtricitabine (which are used together clinically), zidovudine, or abacavir alone. No individual constituent phenocopied the high efficacy of the NRTI cocktail (Fig. 2*A*, columns 7 to 9). However, at higher doses equal to the total dose of the NRTI cocktail, both tenofovir/emtricitabine and abacavir alone significantly reduced abscess formation (Fig. 2*A*, columns 10 and 11). Timing was critical, as NRTI administration at 1 dpi did not prevent abscess formation (Fig. 2*A*, columns 12 and 13). These observations together reveal that preventing abscess formation with NRTIs requires a specific dose administered during a critical window early after infection, the time frame where inflammatory host pathways are triggered (1). Since both tenofovir/emtricitabine and abacavir reduced abscess formation when administered alone, their efficacies are likely due to shared downstream consequences, which may include similar off-target effects or, as we hypothesize, direct inhibition of endogenous reverse transcriptases.

Tenofovir, emtricitabine, and abacavir did not inhibit *E. coli* growth in vitro (Fig. 2*B*). Thus, the efficacy of these drugs alone (Fig. 2*A*, columns 10 and 11) is likely not due to direct bacterial killing. In contrast, zidovudine strongly inhibited *E. coli* growth (Fig. 2*B*). Although zidovudine is present in the NRTI cocktail, zidovudine alone did not reduce abscess susceptibility (Fig. 2*A*, column 8), and therefore, the efficacy of the NRTI cocktail is likely not due to bacterial killing. However, it is possible that NRTIs are uniquely bactericidal in vivo. To further determine whether NRTI efficacy requires bacterial killing, we examined whether NRTIs prevent development of sterile necrotic liver lesions that form following inoculation with formalin-killed bacteria; these lesions are less abundant and morphologically distinct from abscesses containing live *E. coli,* and presumably result from inflammation-induced necrosis without sustained immune cell infiltration following bacterial replication (Fig. 2 *C*, *Middle* row). NRTI treatment prevented the formation of the necrotic lesions (Fig. 2 *C*, *Bottom* row), revealing that NRTIs are modulating the host response to bacterial molecules. Although it is possible that NRTIs heighten bacterial killing in vivo in a manner specific to the use of live bacteria, these data together more likely indicate that NRTIs prevent liver abscesses by inhibiting the necrosis that facilitates initial bacterial replication.

## Concluding Remarks

Here, we demonstrate that NRTIs prevent liver abscess formation during *E. coli* bloodstream infection. We propose that NRTIs inhibit ERV reverse transcription, leading to a reduction in the levels of cytosolic DNA and diminished proinflammatory responses. Since abscesses are tissue-specific and sex-dependent, we speculate that ERV expression and downstream signaling may be regulated in a sex- and tissue-dependent manner. Future work will be targeted toward dissecting how ERVs and NRTIs modulate inflammation.

Silencing ERVs is critical for the host during homeostasis, since the presence of viral epitopes or ERV-mediated insertional mutagenesis can promote autoimmune diseases or cancer (8). In mice, lupus-like phenotypes are associated with polymorphisms in SNERV1/2, which encode suppressors of ERV expression (3). However, during infection, ERVs and potentially other retroelements may amplify inflammation by stimulating immune pathways that recognize nucleic acids. In our model for liver abscess formation, inflammation leads to hepatic necrosis which facilitates bacterial replication. However, in other infection contexts, inflammatory responses mediated by reverse transcription may promote pathogen control. Thus, whether ERVs—and NRTIs—are beneficial or detrimental for the host is likely context-dependent. Together, our data suggest that NRTIs and potentially other antiretrovirals should be reexamined for treatment of other diseases where reverse transcription may drive detrimental inflammatory responses.

## Materials and Methods

Methods details are provided in *SI Appendix*, *Materials and Methods*.

## Supplementary Material

Appendix 01 (PDF)

Dataset S01 (XLSX)

Dataset S02 (XLSX)

## Data Availability

Sequencing reads data have been deposited in SRA (PRJNA952694) (9). All other data are included in the manuscript and/or supporting information.
