# Supplementary material for: Reverse transcriptase inhibitors prevent liver abscess formation during Escherichia coli bloodstream infection
Source: Proc Natl Acad Sci U S A. 2024 Jan 16;121(4):e2319162121. doi: 10.1073/pnas.2319162121 (PMC10823173; doi:10.1073/pnas.2319162121)
Supplement: Supplementary file 1 — Appendix 01 (PDF) [file pnas.2319162121.sapp.pdf]

PNAS

Supporting Information for
Reverse transcriptase inhibitors prevent liver abscess formation during *E. coli* bloodstream infection

Karthik Hullahalli, Katherine G. Dailey, Yuko Hasegawa, Welkin E. Johnson, and Matthew K. Waldor

Address correspondence to KH (hullahalli@g.harvard.edu) or MKW (mwaldor@bwh.harvard.edu)

This PDF file includes:

Materials and Methods
Legends for Dataset S1 and S2

Other supporting materials for this manuscript include the following:

Datasets S1 and S2

Materials and Methods

Ethics. All animal experiments were conducted in accordance with the recommendations in the Guide for the Care and Use of Laboratory Animals of the National Institutes of Health and the Animal Welfare Act of the United States Department of Agriculture using protocols reviewed and approved by Brigham and Women's Hospital Committee on Animals (Institutional Animal Care and Use Committee (IACUC) protocol number 2016N000416 and Animal Welfare Assurance of Compliance number A4752-01).

Animals. 8–12-week-old female mice were used for all experiments. Mice were obtained from Jackson Laboratories (C57BL/6J (B6J, #000664), C57BL/6NJ (B6N, #005304), BALB/cJ (#000651)). Animals were maintained at 68-75ºC with 50% humidity in 12 hour day-night cycles. For infections, defined volumes of frozen CHS7-STAMP library (derived from strain CFT073, isolated from the blood of a patient with pyelonephritis (1, 2)) from overnight cultures were thawed, diluted in PBS, and immediately used to inoculate mice at a dose of $5x10^6$. For injection of killed bacteria, frozen bacteria were resuspended in 4% paraformaldehyde and incubated at room temperature for 20 minutes, washed twice in PBS, and resuspended in PBS. NRTIs were dissolved in PBS and administered at doses indicated by intraperitoneal injection in a total volume of 200µl (Tenofovir disoproxil fumarate: Fisher Scientific AC461250010, Emtricitabine: Sigma PHR2120-500MG, Zidovudine: Sigma PHR1292-1G, Abacavir: Fisher Scientific AC458860010). For intravenous injections, animals were restrained using a Broome-style restrainer (Plas-Labs) and inoculated via the lateral tail vein with 100µl using a 27G needle. A heating pad was used to facilitate dilation of the tail vein. For intraperitoneal injections, animals were injected with 200µl into the abdominal cavity with a 25G needle. Animals were euthanized by isoflurane inhalation and cervical dislocation. To quantify CFU, organs were excised and homogenized with 2 x 3.2 mm stainless steel beads for 2 minutes with a bead beater (BioSpec). Organs were plated and diluted on LB + 50µg/ml Kanamycin. Abscesses were defined as the visible appearance of at least one white lesion and CFU > 10^4, criteria established and used in our previous study (3).

RNA Sequencing. BALB/cJ, B6J, and B6N females were infected IV and livers were immediately flash frozen in liquid nitrogen 4 hours post inoculation. Total RNA was extracted with the Direct-zol RNA miniprep kit (Zymo) and library preparation was performed with the NEB Ultra II Directional RNA Library Prep Kit (New England Biolabs). Libraries were sequenced on a NovaSeq 6000 instrument at Harvard Medical School Biopolymers Core Facility as 1x101nt reads. Reads were trimmed with TrimGalore using default settings and mapped using HISAT2 to the GRCm38 (mm10) mouse genome. FeatureCounts (4) was used to quantify transcript abundance. Importantly, only uniquely mapped reads were counted, and therefore highly repetitive loci with no unique sequences were excluded. ERV loci were counted using .gtf files from the Genome-based Endogenous Viral Element (gEVE) database (5). Counts were analyzed using DESeq2 for differential expression analysis (6). To identify infection-induced transcripts that correlated with abscess frequency, we quantified differentially regulated genes with adjusted P-value less than 0.05 and log2 fold change greater than 1, between infected B6J vs infected BALB/cJ, infected B6N vs infected B6J, and infected B6N vs uninfected B6N.

Growth Curves. Approximately ~10^8 CFU were collected from *E. coli* colonies on LB plates and resuspended in 10ml of LB. Bacterial suspensions were diluted 1/3 in NRTIs with indicated concentrations in LB and cultured overnight with shaking in a microplate reader (BioTek). OD600 was monitored every 10 minutes.

Sequence analysis. Nucleotide sequences correspond to MLV genomes and MLV-related ERV loci were aligned using CLUSTAL-omega. A segment corresponding to the start of the *gag* gene through the end of the *env* gene (relative to Moloney MLV) was extracted and used to identify the best-fit tree by maximum likelihood using PHYML, as implemented in Geneious Prime (2023.1.2).

The tree image was created in FigTree (v.1.4.4). HEMV, an ERV from *Mus spiceligus*, was designated as an outgroup based on previously reported relationship to MLVs (7).

Data availability. RNA-Sequencing reads have been deposited to the Sequencing Read Archive (PRJNA952694). Read count tables are provided as Dataset S1 and Dataset S2.

Legends for supporting datasets.

Dataset S1 (separate file). Table of read counts for non-endogenous retroelement loci. Raw read counts are shown for GENCODE M25 (GRCm38) annotations.

Dataset S2 (separate file). Table of read counts from ERV loci. Raw read counts are shown for gEVE (GRCm38) annotations.

SI References

1. Welch RA, Burland V, Plunkett G, Redford P, Roesch P, Rasko D, Buckles EL, Liou S-R, Boutin A, Hackett J, Stroud D, Mayhew GF, Rose DJ, Zhou S, Schwartz DC, Perna NT, Mobley HLT, Donnenberg MS, Blattner FR. 2002. Extensive mosaic structure revealed by the complete genome sequence of uropathogenic Escherichia coli. Proc Natl Acad Sci U S A 99:17020–4.
2. Hullahalli K, Waldor MK. 2021. Pathogen clonal expansion underlies multiorgan dissemination and organ-specific outcomes during murine systemic infection. Elife 10:e70910.
3. Hullahalli K, Dailey KG, Hasegawa Y, Suzuki M, Zhang H, Threadgill DW, Waldor MK. 2023. Genetic and immune determinants of E. coli liver abscess formation. bioRxiv 543319.
4. Liao Y, Smyth GK, Shi W. 2014. featureCounts: an efficient general purpose program for assigning sequence reads to genomic features. Bioinformatics 30:923–30.
5. Nakagawa S, Takahashi MU. 2016. gEVE: a genome-based endogenous viral element database provides comprehensive viral protein-coding sequences in mammalian genomes. Database (Oxford) 2016.
6. Love MI, Huber W, Anders S. 2014. Moderated estimation of fold change and dispersion for RNA-seq data with DESeq2. Genome Biol 15:550.
7. Tipper CH, Bencsics CE, Coffin JM. 2005. Characterization of hortulanus endogenous murine leukemia virus, an endogenous provirus that encodes an infectious murine leukemia virus of a novel subgroup. J Virol 79:8316–29.
